# Characterization of Enlarged Tongues in Cloned Piglets

**DOI:** 10.3390/cimb45110571

**Published:** 2023-11-14

**Authors:** Mi-Ryung Park, Jin Seop Ahn, Min Gook Lee, Bo Ram Lee, Sun A Ock, Sung June Byun, In-Sul Hwang

**Affiliations:** 1 Animal Biotechnology Division, National Institute of Animal Science, Rural Development Administration, Wanju 55365, Republic of Korea; mrpark45@korea.kr (M.-R.P.);; 2 Columbia Center for Translational Immunology, Columbia University Irving Medical Center, Columbia University, New York, NY 10032, USA

**Keywords:** transgenic pig, SCNT, developmental defect, transcriptome

## Abstract

Although the efficiency of cloning remains very low, this technique has become the most reliable way to produce transgenic pigs. However, the high rate of abnormal offspring such as an enlarged tongue lowers the cloning efficiency by reducing the early survivability of piglets. Thus, the present study was conducted to identify the characteristics of the enlarged tongue from cloned piglets by histologic and transcriptomic analysis. As a result, it was observed that the tissues from enlarged tongues (*n* = 3) showed isolated and broken muscle bundles with wide spaces while the tissues from normal tongues (*n* = 3) showed the tight connection of muscle bundles without space by histological analysis. Additionally, transmission electron microscopy results also showed the formation of isolated and broken muscle bundles in enlarged tongues. The transcriptome analysis showed a total of 197 upregulated and 139 downregulated genes with more than 2-fold changes in enlarged tongues. Moreover, there was clear evidence for the difference between groups in the muscle system process with high relation in the biological process by gene ontology analysis. The analysis of the Kyoto Encyclopedia of Gene and Genomes pathway of differentially expressed genes indicated that the pentose phosphate pathway, glycolysis/gluconeogenesis, and glucagon signaling pathway were also involved. Conclusively, our results could suggest that the abnormal glycolytic regulation may result in the formation of an enlarged tongue. These findings might have the potential to understand the underlying mechanisms, abnormal development, and disease diagnosis in cloned pigs.

## 1. Introduction

The importance of pigs in studying biomedical and agricultural research related to human disease and life has gradually increased. Because the anatomical and physiological similarities between pigs and humans allow substantial application of pigs as model animals for drug research, toxicological research, disease research, and xenotransplantation research [1,2,3,4]. Usually, genome editing with somatic and reproductive cells can be applied to generate temporal and permanent transgenic pigs [3,5].

To produce permanent transgenic pigs, mainly two methods such as microinjection and somatic nuclear transfer (SCNT) were developed decades ago and are commonly used to the present depending on the purpose of application [6,7]. In case of the SCNT method has some advantages compared to the microinjection method including non-mosaicism and germline transmission while this method has some disadvantages including very low efficiency and developmental defects [8]. Especially, the low efficiency and developmental defects can occur throughout the gestation period and before and after farrowing [7]. The reasons for these abnormalities are unknown but may be caused by incomplete and/or inappropriate reprogramming of donor cells related to problems during imprinting [9]. Also, some reports suggested that incomplete epigenetic reprogramming of the donor cell causes developmental defects resulting in low efficiency of SCNT [10,11,12]. Many kinds of symptoms from cloned pigs were reported including over-weight, giant forelimbs, atrophy of tendons, enlarged tongue, and exomphalos [13,14,15,16]. Among these, the incidence of abnormally enlarged tongues is higher compared to other symptoms, but the causes and mechanisms of its occurrence have not yet been fully understood [14,16,17]. 

Despite these disadvantages, there were many trials to produce effective recombinant drugs from the mammary gland of transgenic animals by the SCNT method. For example, a kind of recombinant drug for the treatment of cerebral ischemic stroke such as the human tissue-type plasminogen activator (htPA) is still challenge to produce from transgenic animals including mice [18], rabbits [19], goats [20], and pigs [21]. Because transgenic animals can offer advantages over traditional methods involving cell cultures or bacteria, such as lower cost, higher biological activity, and the ability to obtain fully translated proteins [22]. 

Therefore, the present study was conducted to produce htPA transgenic pigs and investigate the characteristics of enlarged tongues from cloned piglets by histologic and transcriptomic analysis. In addition, the developmental competence of cloned embryos using two different transgenic cell lines and the production status of cloned piglets were analyzed.

## 2. Materials and Methods

### 2.1. General Information and Ethycal Statement

All chemicals used in the present study were purchased from Sigma-Aldrich Chemicals (St. Louis, MO, USA) unless otherwise stated. The procedures and standard operating protocols for the treatment of the pigs were reviewed and approved by the Institutional Animal Care and Use Committee of the National Institute of Animal Science, RDA (approval no. NIAS2015-736, 1 March 2015). 

### 2.2. Experimental Design

Briefly, slaughterhouse-derived oocytes were matured in vitro, and subjected to the SCNT procedures. In experiment 1, the developmental competence of cloned embryos with 2 different transgenic htPA cells was analyzed in vitro culture for 7 days. In experiment 2, cloned embryos were transferred into surrogate mothers to produce transgenic piglets. The production status of htPA piglets was investigated including cloning efficiency, litter size, weight of newborn piglets, and so on. Finally, in experiment 3, characteristics of piglets that show symptoms of enlarged tongue were investigated including histologic and transcriptomic analysis. 

### 2.3. In Vitro Maturation

The in vitro maturation (IVM) protocol applied in the present study was adopted from our previous study [23]. Briefly, the ovaries from prepubertal gilts were collected from a local slaughterhouse (Nonghyup Moguchon, Gimje, Korea) and transported to the laboratory at about 35 °C in 0.9% saline within 4 h. The follicular fluid with cumulus-oocyte complexes (COCs) was aspirated from follicles (3–6 mm in diameter) and washed three times in Medium-199 (ThermoFisher Scientific, Waltham, MA, USA) supplemented with 0.1% (*w*/*v*) polyvinyl alcohol (PVA). Then, the COCs were matured in 500 µL Medium-199 containing 0.1% polyvinyl alcohol (*w*/*v*), 3.05 mM D-glucose, 0.91 mM sodium pyruvate, 0.57 mM cysteine, 0.5 µg/mL luteinizing hormone, 0.5 µg/mL follicle stimulating hormone, 10 ng/mL epidermal growth factor, 10% porcine follicular fluid (*v*/*v*), 75 µg/mL penicillin G, and 50 µg/mL streptomycin (maturation medium). COCs were matured for 22 h in the maturation medium and another 22 h in the maturation medium without luteinizing and follicle-stimulating hormones at 38.5 °C under 5% CO_2_ in the air. After 44 h of IVM, cumulus cells were removed by gentle pipetting after treatment of COCs with 0.1% hyaluronidase for 5 min. 

### 2.4. Production of Transgenic Cell Lines

The construction of the htPA expression vector followed the previously described method [24]. We obtained porcine ear fibroblast (PEF) from the crossbred piglet (Male, 3 months old, Landrace x Yorkshire) cultured in Dulbecco’s modified Eagle’s medium (ThermoFisher Scientific) containing 20% fetal bovine serum, and 1% antibiotics (100 U/mL penicillin and 100 mg/mL streptomycin; Life Technologies, Carlsbad, CA, USA) at 37 °C in an atmosphere of 5% CO_2_. The ear fibroblasts were transfected with 4 ug of a htPA expression vector using the Amaxa nucleofector with the U-023 program (Lonza, Basel, Switzerland). After, transfected cells were selected by neomycin (ThermoFisher Scientific) to culture a single colony. To verify the transgenic cell lines, PCR was conducted using genomic DNA and the primers listed in Table S1. PCR cycles were as follows: initial denaturation at 95 °C for 5 min; by 40 cycles of 94 °C for 30 s, 60 °C for 30 s, and 72 °C for 1 min (for 0.3 kb), 2 min (for 2.3 kb), or 6 min (for 6.6 kb); followed by a final extension at 72 °C for 5 min. beta-actin was used as the internal control.

### 2.5. Somatic Cell Nuclear Transfer

Somatic cell nuclear transfer with transgenic htPA cells was performed based on our previous reports [23,25,26]. Matured oocytes with visible first polar were enucleated by aspirating the first polar body and adjacent cytoplasm containing metaphase II chromosomes using a beveled pipette-sized 16–18 µm diameter. Then, transgenic cells were injected into the perivitelline space of enucleated oocytes. The oocytes injected transgenic cells were placed into 0.2-mm diameter electrodes of a fusion chamber filled with 0.3 M mannitol solution consisting of 0.1 mM MgSO_4_, 1.0 mM CaCl_2,_ and 0.5 mM Hepes. For fusion, 2 DC pulses of 1.5 kV/cm were applied for 30 µs using a cell fusion generator (Nepa Gene Co., Ichikawa, Chiba, Japan). After an hour of incubation in porcine zygote medium (PZM)-3 [27] containing 0.3% (*w*/*v*) bovine serum albumin (BSA), successfully cloned embryos were selected to conduct further experiments such as in vitro culture and embryo transfer.

### 2.6. In Vitro Culture and Embryo Transfer

For in vitro culture, the cloned embryos were transferred into a 4-well dish (ThermoFisher Scientific) containing PZM-3 with 0.3% (*w*/*v*) BSA. In vitro culture for 7 days was carried out in an incubator at 38.5 °C under 5% CO_2_ in air. For determination of developmental competence in vitro, the cleavage rate and blastocyst formation rate were recorded on days 2 and 7 of culture, respectively. Immediately after the confirmation of fusion, the cloned embryos were transferred into the oviducts of the recipients on the same day or 1 day after the onset of estrus to produce transgenic htPA piglets. In total, 28 surrogate mothers were applied for embryo transfer to establish pregnancy. Then, all surrogate mothers had an ultrasound diagnosis routinely on day 27 to confirm gestation sac formation, day 35 to confirm maintenance of pregnancy, and day 97 to confirm fetus formation. All cloned piglets were delivered by natural farrowing. 

### 2.7. Histology of Tongue Tissues

Both tongue tissues from cloned and wild-type piglets were fixed in 10% buffered formalin solution and conducted histological analysis. After fixation, all tissues were washed three times in PBS and dehydrated from 50 to 100% ethanol solutions by stages. After paraffin embedding, the tissues were sliced (4 μm thickness) and stained with hematoxylin and eosin for general analysis purposes. For analysis of a transmission electron microscope (TEM, Hitachi, Japan), the cloned piglets’ tongue tissue blocks were fixed in pre-chilled 2% glutaraldehyde at 4 °C for two hours. Then, the tissue was rinsed three times in 0.05 M sodium cacodylate buffer (pH 7.2) at 4 °C for 10 min. The blocks were dehydrated in a stepwise manner from 30 to 100% gradient alcohol at room temperature. The tissue blocks were then embedded into epoxy resin to form small spheres. The embedded tissue blocks were randomly selected to slice into ultrathin sections of 80 nm thickness by Ultramicrotome (EM UC7, Leica, Wetzlar, Germany). Finally, the slices were double stained with 2% uranyl acetate and Reynold’s lead citrate, followed by a wash in distilled water for removal of stain residues. 

### 2.8. Genomic Analysis

The total RNA was extracted using TRIzol reagent from tongue tissues, following the manufacturer’s instructions. To prevent genomic DNA contamination, DNase I treatment was applied to the RNA samples. The quality of the RNA was evaluated, ensuring that the RNA Integrity Number (RIN) was greater than 7 by utilizing the Agilent Technologies 2100 Bioanalyzer. Subsequently, sequencing libraries were generated from 50 ng of tissue samples and sequenced using the Tru-seq system (Macrogen, Seoul, Korea). Raw RNA sequencing (RNA-Seq) reads were filtered, and the Sus scrofa reference genome (NCBI.Sscrofa11.1) was obtained by Tophat (version 2.0.13) and Bowtie2 (version 2.2.3). The assembly of transcript models from the alignments and the estimation of their abundance in the transcriptome was performed using Cufflinks (version 2.2.1). The transcript abundance was improved by quantile normalization and correction of sequence bias to enhance expression estimates [28]. Differentially expressed genes (DEGs) were identified based on the following criteria: FDR-adjusted *p*-value < 0.05 and an absolute log2-fold change > 1. The fold change was calculated as the expression in the cloned piglet samples divided by the expression in the wild-type piglets. Hierarchical cluster analysis of the DEGs was carried out using MultiExperiment Viewer, and Gene Ontology (GO) analysis was performed. The pathway analysis was performed using the online tool ShinyGO, the Kyoto Encyclopedia of Gene and Genomes (KEGG), and the Database for Annotation, Visualization, and Integrated Discovery (DAVID).

### 2.9. Statistical Analysis

All data were analyzed by use of the SAS Enterprise Guide 7.1 (SAS Institute Inc., Cary, NC, USA). The developmental competence of cloned embryos in groups was compared by the ANOVA, followed by the *t*-test. The data are expressed as mean ± standard error of the mean (SEM). The significant difference was set at *p* < 0.05.

## 3. Results

### 3.1. Establishment of Transgenic Cell Lines

**Figure 1:**
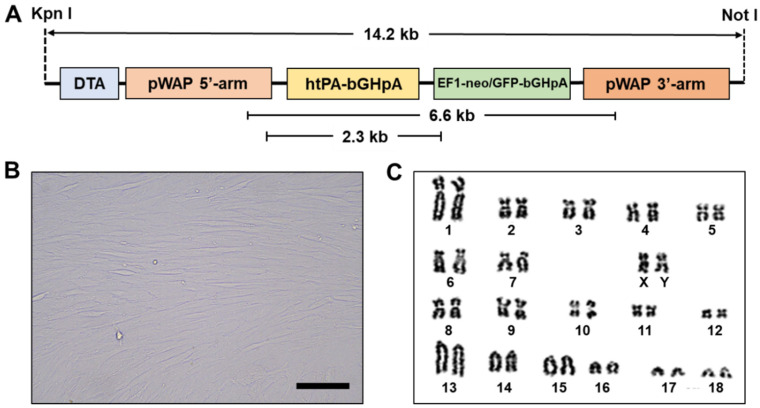
Construction of htPA expression vector and establishment of htPA transgenic cell lines. (**A**) Structure of htPA expression vector. (**B**) Representative image of htPA transgenic cells. (**C**) Representative image of karyotyping results from htPA transgenic cells. Bar = 100 μm.

For the establishment of the htPA transgenic cell line, male porcine ear fibroblast cells (3 months old, crossbred of Landrace x Yorkshire) were transfected with a targeting vector constructed as shown in Figure 1A. A mammary-specific expression vector was constructed in which 5’ sequences from the whey acid protein (WAP) gene were added with a cDNA coding for htPA activator while neomycin/GFP and Diphtheria toxin A (DTA) were inserted as a positive selection marker and negative marker, respectively. After transfection, neomycin-resistant colonies were obtained and proliferated as shown in Figure 1B. Two transgenic cell lines (#3 and #4) were selected for the production of transgenic pigs after confirmation of no abnormality by karyotyping analysis (Figure 1C).

### 3.2. Developmental Competence of Cloned Embryos

**Figure 2:**
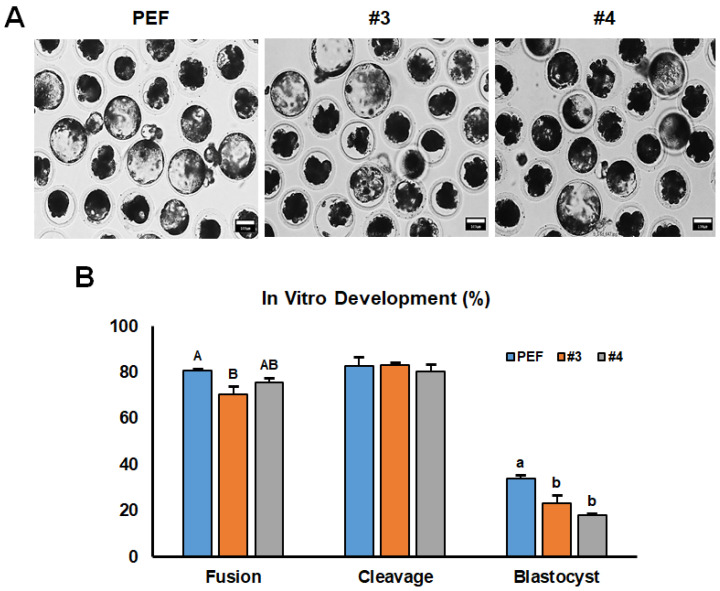
Developmental characteristics of cloned htPA embryos depending on donor cell lines in vitro. (**A**) Representative morphology of cloned htPA embryos. (**B**) Developmental competence of cloned htPA embryos. ^A,B and a,b^ Different superscripts indicate significant differences between PEF and transgenic cell line in panel B (*p* < 0.05). Bars = 100 μm.

As shown in Figure 2A, three groups of cloned embryos were generated with #3, #4, and PEF cells as donors. The morphology of each blastocyst from the three groups was comparable while the fusion rate in the #3 group (70.3%) was lower significantly (*p* < 0.05) than other two groups (#4; 75.6% and PEF; 80.7%) as shown in Figure 2B. Although there was no significant difference in cleavage rate between all groups (#3; 83.0%, #4; 80.3%, PEF; 82.7%), the blastocyst formation rates were decreased significantly in groups #3 (23.1%) and #4 (18.1%) compared to the PEF group (33.9%). 

### 3.3. Production of Cloned htPA Transgenic Piglets

As shown in Table 1, a total of 9145 cloned embryos were produced by the SCNT method using 2 different transgenic cell lines. All 3687 cloned embryos were transferred to 11 surrogate mothers with a mean number of 335.2 in the #3 group while all 5458 cloned embryos were transferred to 17 surrogate mothers with a mean number of 321.1 in the #4 group. Seven of twenty-eight surrogate mothers delivered successfully with 23 piglets and the litter size between groups #3 and #4 was comparable. However, the delivery rate of the #3 group showed a higher tendency than the #4 group. The cloning efficiencies were 0.3% and 0.2% in the #3 and #4 groups, respectively. The weight of newborn piglets in the #3 group (964.1 g) was significantly larger (*p* < 0.05) than the #4 group (826.7 g).

**Figure 3:**
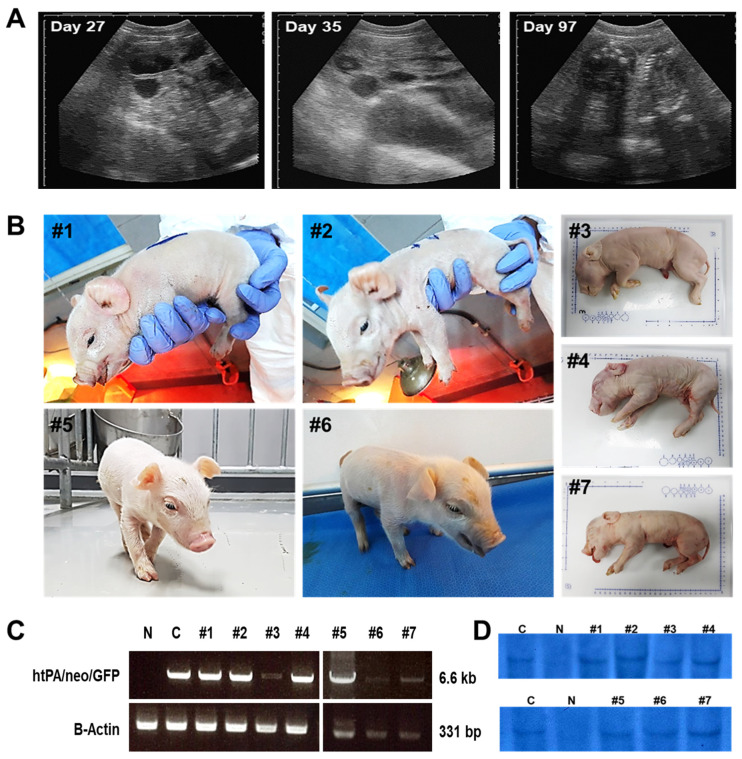
Confirmation of cloned htPA piglets. (**A**) Representative pregnancy diagnosis by ultrasound on days 27, 35, and 97. (**B**) Representative litter sizes of the #3 group (#1 to #4) and #4 group (#5 to #7). (**C**) Confirmation of transgene expression by PCR. (**D**) Confirmation of transgene expression by Southern blotting analysis. N; PEF cells (wild type), C; htPA transfected donor cells, #1 to #7; tissues from cloned piglets. The #1, #6, and #7 piglets have macroglossia-like phenotypes.

As shown in Figure 3A, all pregnant surrogate mothers had an ultrasound diagnosis routinely on day 27 to confirm gestation sac formation, on day 35 to confirm maintenance of pregnancy, and on day 97 to confirm fetus formation. A total of 7 surrogate mothers delivered offspring, and 7 out of 23 offspring were stillborn (Figure 3B and Table 1). Figure 3B shows the representative litter size and morphology of newborn piglets; #1 to 4 were delivered by a surrogate mother that was transferred #3 cell-line-derived cloned embryos, and #5 to 7 were delivered by a surrogate mother that was transferred #4 cell line-derived cloned embryos. Some of the piglets showed symptoms of macroglossia-like phenotypes (piglets #1, #6, and #7). The tongues of these symptomatic piglets were collected for further analysis. However, all these newborn piglets were confirmed as htPA transgenic pigs by PCR and Southern blotting analysis (Figure 3C,D). Additionally, microsatellite analysis indicated that these piglets were derived from htPA transgenic cells (Table S2).

### 3.4. Histological Analysis of Abnormal Tongues in Cloned htPA Piglets

**Figure 4:**
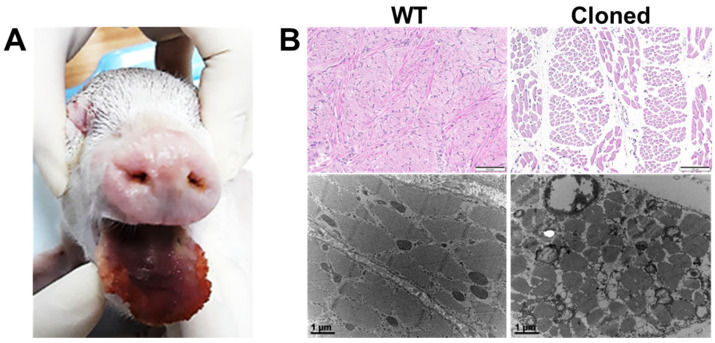
Histological images of abnormal tongue tissues in cloned piglets. (**A**) The phenotype of the abnormal macroglossia-like tongue. (**B**) Representative images after hematoxylin and eosin staining (upper) and transmission electron microscopy (lower) of tongue tissues. WT: tissues from wild-type piglets, Cloned: tissues from cloned piglets. Bars = 100 μm (upper) and 1 μm (lower).

We found that cloned htPA piglets showed abnormal phenotype in the tongue (Figure 4A). As mentioned previously, those abnormal tongues were like that of the phenotype of macroglossia. Thus, we collected specimens from both cloned and wild-type piglet tongues to analyze histological differences. As shown in Figure 4B, it was observed that the tissues from normal tongues showed a tight connection of muscle bundles without space while the tissues from enlarged tongues showed isolated and broken muscle bundles with wide spaces by histology. The result of transmission electron microscopy also showed the formation of isolated and broken muscle bundles in enlarged tongues. 

### 3.5. Analysis of Transcriptome of Abnormal Tongue in Cloned Piglets

**Figure 5:**
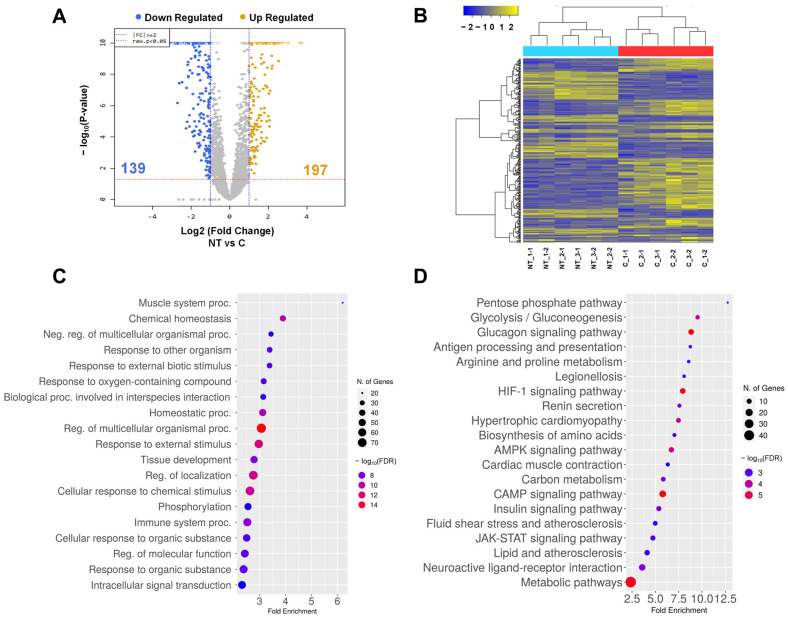
Transcriptome analysis of tongue tissues from cloned piglets. (**A**) Volcano plotting analysis of 197 upregulated genes and 139 downregulated genes. (**B**) Heat map analysis from the one-way hierarchical clustering of up and down-regulated DEGs using the Euclidean method and complete linkage. (**C**) Gene ontology (GO) analysis to identify biological processes. (**D**) KEGG pathway analysis. The results of GO and KEGG analysis were carried out by using the ShinyGO 0.77 (http://bioinformatics.sdstate.edu (accessed on 1 March 2023)) with selection by FDR and sorting by fold enrichment. C; wild type, NT; cloned piglet, Neg; negative, Proc; process, Reg; regulation.

To clarify the cause of the phenomenon mentioned previously, transcriptome analysis was applied using the RNA-seq technique. For transcriptome analysis, RNA was isolated from tongue tissues from wild type and cloned piglets. In total, 197 upregulated genes and 139 downregulated genes with more than two-fold change were identified (Figure 5A, Tables S3 and S4). Also, there was a clear difference between wild-type and cloned piglets confirmed by heat map analysis with a one-way hierarchical clustering python of a total of 336 genes (Figure 5B). The GO analysis results in the biological process indicated a strong association with the muscle system process and chemical homeostasis. (Figure 5C and Table S5). The KEGG pathway analysis results were highly associated with glycolytic-related pathways such as the pentose phosphate pathway, glycolysis/gluconeogenesis, and the glucagon signaling pathway (Figure 5D and Table S6). 

**Figure 6:**
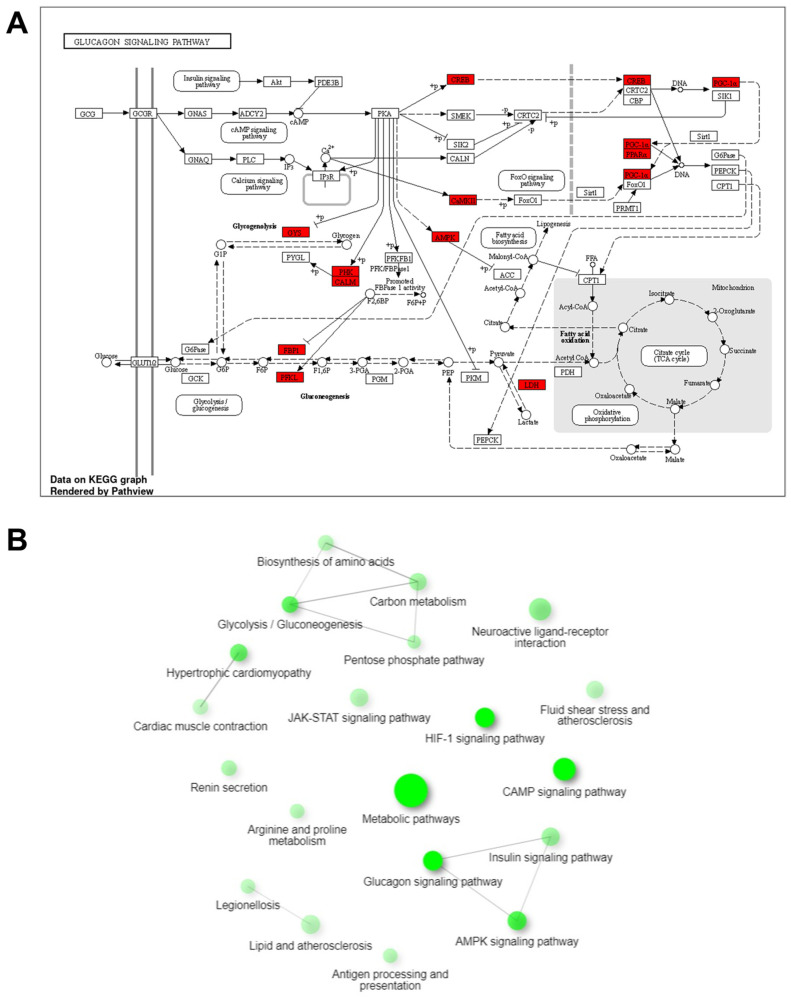
Analysis of KEGG pathway and interactive enrichment networks. (**A**) The glucagon signaling pathway. (**B**) Analysis of interactive enrichment networks between differentially expressed genes. The red rectangles indicate the DEGs. The edge cut was 0.3 value.

As shown in Figure 6A, DEGs highlighted in red rectangle were observed to be associated with the glucagon signaling pathway. The interactive enrichment network was examined using the results from the KEGG pathway analysis (Figure 6B). The glucagon signaling pathway was found to intersect significantly with the insulin, glucagon, and adenosine monophosphate-activated protein kinase signaling pathways, along with strong interactions with pentose phosphate, amino acid biosynthesis, glycolysis, and carbon metabolism.

## 4. Discussion

In the present study, we generated a transgenic cell line with a normal karyotype that expresses htPA using a WAP promoter. It is well known that the htPA activates the dissolution of cellulose that makes blood clots to be broken down and helps supply and circulation blood [29]. Thus, recombinant tPA is currently used as the standard treatment for acute ischemic stroke, pulmonary embolism, and myocardial infarction [30,31]. Therefore, many attempts to generate the htPA transgenic animals, as those in the present study, were conducted in many species including mice [32], rats [33], goats [34], cows [35], rabbits [18], and pigs [21]. The WAP promoter applied in the present study (Figure 1A) is a mammary gland-specific promoter that of the WAP protein regulates the proliferation of mammalian epithelial cells [36]. The WAP protein is specifically expressed in the mammary gland and is controlled by hormones and developmental signals during pregnancy [37]. Many studies have confirmed that the mammary glands of transgenic animals can serve as bioreactors to produce valuable therapeutic proteins [38,39,40].

We described decreased formation rates of the blastocyst in the experimental groups (23.1% and 18.1%) than the control group (33.9%). These lower rates of blastocyst formation were likely due to the characteristics of the transgenic donor cells and were not significantly different from those of previous reports (ranging from 15% to 30%) using transgenic donor cells for the SCNT [41,42,43]. After 28 times of embryo transfers, a total of 23 piglets were delivered with 7 stillborn (30.0%) from 28 surrogate mothers which is slightly higher than a previous report (23.6%) by Kurome et al. [44]. It is well known that there are many kinds of factors influencing the outcome of cloning in pigs including season, type of gene modification, donor cell source, cloning rounds, and selection of cloned embryos for early development [44]. In the present study, we could confirm the different tendencies of developmental competence of cloned embryos between two different cell lines which could be an influencing factor. However, among them, three piglets exhibited macroglossia-like symptoms. Therefore, we analyze the underlying causes of symptoms in cloned piglets. 

As described above in the introduction section, one of the reasons for decreasing the efficiency of cloning in pigs is developmental defects such as being overweight, giant forelimbs, atrophy of tendons, enlarged tongue, and exomphalos [13,14,15,16]. In the present study, we found some developmental defects including abnormal front legs, enlarged tongue, and hernia. We decided to analyze only enlarged tongue because that was the highest prevalence rate in a previous report by Yang et al. [16]. As shown in Figure 4, we confirmed for the first time that the enlarged tongue from a cloned piglet exhibits very strange patterns of broken and isolated muscle bundles. Thus, we decided to analyze the pathway related to abnormal development using RNA-seq. 

Additionally, it is widely recognized that cloned pigs exhibit various external and internal malformations, such as kyphosis, lordosis, digit and kidney agenesis, malformed ears, clefts, macroglossia (enlarged tongue), and heart defects [17]. As described in the present study, we focused on analyzing the characteristics of the enlarged tongue such as macroglossia during the procedures of transgenic pig production. The macroglossia in cloned pigs is the most common malformation regardless of transgene types, expression level, and pig breed [17]. However, when the piglets show macroglossia-like symptoms, it is very difficult to manage their health because the enlarged tongue makes the suckling ability of newborn piglets very difficult. Thus, to increase the cloning efficiency and viability of piglets, the abnormal piglets should receive intensive care with artificial nursing. 

Interestingly, we found that abnormal tongue development of cloned piglets appeared to be differential expressions of transcripts related to the pentose phosphate pathway (PPP), glycolysis/gluconeogenesis, and glucagon signaling pathway. This indicates that the abnormal expressions in those pathways directly related to glucose synthesis occurred in the cloned piglets. This way of analysis can reflect the cause and result of abnormal phenotypes in cloned piglets. The PPP is a metabolic pathway that is responsible for producing NADPH and pentoses from glucose [45,46]. Furthermore, it has shown a strong association with glycolysis/gluconeogenesis, which are essential mechanisms for glucose synthesis and inhibition. Aberrant regulation of these processes can potentially lead to the development of Glycogen Storage Diseases (GSD) [47]. Additionally, a correlation was observed with the glucagon signaling pathway, which stimulates gluconeogenesis to raise blood glucose levels [48]. The Beckwith–Wiedemann syndrome (BWS), a disorder characterized by overgrowth, presents symptoms such as macroglossia and macrosomia in the neonatal period and embryonal cancers of infancy. It is associated with hypoglycemia at a frequency of 30–50%. Therefore, abnormalities in glucose can lead to diseases associated with abnormal growth [49]. Thus, it is possible that the glucose-associated pathway disrupted in the present study resulted in abnormal phenotypes of cloned piglets. Moreover, it is well known that oxidative PPP is required for vascular maturation to control the expression of elastin through the production of ribose-5-phosphate and deposition in endothelial cells [50]. The abnormal glycolysis/gluconeogenesis generated by disrupted pathways may cause glycogen storage disorder resulting in diseases in the liver, muscles, and brain [51]. As a result, the GO terms analysis of the biological process revealed that the muscle system process exhibited the highest fold change value and associated. Overall, it can be concluded that the enlarged tongue in cloned pigs may be influenced by the abnormal development of tongue muscle tissue and multiple glucose-related pathways.

## 5. Conclusions

In this study, the cloned transgenic pigs were generated through the SCNT technique. As a result, 3 out of the 23 piglets exhibited macroglossia-like symptoms. The histological analysis showed that abnormal longue tissues exhibited broken and isolated muscles. Moreover, the transcriptome analysis showed abnormalities in glucose-related pathways. This can lead to diseases such as GSD and BWS. Specifically, BWS exhibits symptoms such as macroglossia, with a high frequency of hypoglycemia during the neonatal period. Therefore, the abnormal development of the tongue in cloned animals is likely to occur similarly. Through future research, we will clarify the relationship between the occurrence of macroglossia and transgenes, as well as the functionality through studies related to glucose pathways Overall, this study provided understanding into the causes of abnormal macroglossia in cloned pigs through transcriptome analysis and results from histological analysis. The findings described in this study have the potential to offer valuable insights into the underlying mechanisms, abnormal development, and disease diagnosis in cloned animals. 

## Figures and Tables

**Table 1:** Production of cloned htPA transgenic piglets.

Donor Cells		#3	#4	Total
No. of embryos transferred				
	Total	3687	5458	9145
	Mean ± SD	335.2 ± 20.5	321.1 ± 12.9	326.6 ± 11.1
No. of surrogates				
	Total	11	17	28
	Delivered (%)	4 (36.4)	3 (17.7)	7 (25.0)
No. of offspring (stillborn)		11 (4)	12 (3)	23 (7)
No. of offspring/embryos (%)		11/3687 (0.3)	12/5458 (0.2)	ND
Mean weight of offspring (g) *		964.1 ± 70.1 ^a^	826.7 ± 58.1 ^b^	ND

* Body weights of piglets are expressed as means ± SEM. ^a and b^ Different superscripts indicate significant differences (*p* < 0.05).

## Data Availability

The data presented in this study are available on request from the corresponding author.

## Supplementary Materials

The following supporting information can be downloaded at: https://www.mdpi.com/article/10.3390/cimb45110571/s1. Table S1: List of primer sequences for PCR; Table S2: Microsatellite analysis of surrogate, donor cell, and offspring; Table S3: Upregulated DEGs between wild type and cloned piglets; Table S4: Downregulated DEGs between wild type and cloned piglets; Table S5: Analysis of enriched biological process GO Terms for DEGs; Table S6: Analysis of enriched KEGG pathway for DEGs.
