# Supplementary material for: Characterization of Enlarged Tongues in Cloned Piglets
Source: Curr Issues Mol Biol. 2023 Nov 14;45(11):9103–16. doi: 10.3390/cimb45110571 (PMC10670481; doi:10.3390/cimb45110571)
Supplement: Supplementary file 1 [file cimb-45-00571-s001.zip › Table S1.pdf]

Table S1. List of primer sequences for PCR

Name primer	Sequences (5' → 3')	Expected size
pWAP 5`& EF1-Neo/GFP	F: CATCTGGGGTCTATCCAAGC<br>R: CACGGCGACTACTGCACTTA	2.4kb
pWAP 5` & pWAP 3`	F: GATGGGAAGAAGTGAGGGGCTCTGTGTC<br>R: GGTACACAGGGGCTAATAGGCAGTCGAG	6.6kb
β-actin	F: GGACTTCGAGCAGGAGATGG<br>R: GCACCGTGTTGGCGTAGAGG	0.3kb
