# Supplementary material for: Characterization of Enlarged Tongues in Cloned Piglets
Source: Curr Issues Mol Biol. 2023 Nov 14;45(11):9103–16. doi: 10.3390/cimb45110571 (PMC10670481; doi:10.3390/cimb45110571)
Supplement: Supplementary file 1 [file cimb-45-00571-s001.zip › Table S2.pdf]

Table S2. Microsatellite analysis of surrogate, donor cell and offspring.

No. of MS markers	Surrogate mother		Donor cell		TG #1		TG #2		TG #3		TG #4		TG #5		TG #6		TG #7	
	Peak 1	Peak 2	Peak 1	Peak 2	Peak 1	Peak 2	Peak 1	Peak 2	Peak 1	Peak 2	Peak 1	Peak 2	Peak 1	Peak 2	Peak 1	Peak 2	Peak 1	Peak 2
S0227	239	239	227	251	227	251	227	251	227	251	227	251	227	251	227	251	227	251
SW2410	106	118	106	116	106	116	106	116	106	116	106	116	106	116	106	116	106	116
SW107	196	196	178	188	178	188	178	188	178	188	178	188	178	188	178	188	178	188
SW1920	128	128	128	130	128	130	128	130	128	130	128	130	128	130	128	130	128	130
SW68	211	249	227	245	227	245	227	245	227	245	227	245	227	245	227	245	227	245
SW210	237	237	235	237	235	237	235	237	235	237	235	237	235	237	235	237	235	237
SW403	101	117	103	103	103	103	103	103	103	103	103	103	103	103	103	103	103	103
SW226	176	190	178	178	178	178	178	178	178	178	178	178	178	178	178	178	178	178
SW414	144	144	144	144	144	144	144	144	144	144	144	144	144	144	144	144	144	144
SW018	248	248	250	258	250	258	250	258	250	258	250	258	250	258	250	258	250	258
SW316	161	161	147	153	147	153	147	153	147	153	147	153	147	153	147	153	147	153
SW1377	206	208	206	208	206	208	206	208	206	208	206	208	206	208	206	208	206	208
SW2456	190	190	190	208	190	208	190	208	190	208	190	208	190	208	190	208	190	208
SW1941	212	218	214	218	214	218	214	218	214	218	214	218	214	218	214	218	214	218
SW2515	93	93	93	95	93	95	93	95	93	95	93	95	93	95	93	95	93	95
SW2401	157	159	161	165	161	165	161	165	161	165	161	165	161	165	161	165	161	165
SW1989	242	244	240	242	240	242	240	242	240	242	240	242	240	242	240	242	240	242
