# Supplementary material for: Characterization of Enlarged Tongues in Cloned Piglets
Source: Curr Issues Mol Biol. 2023 Nov 14;45(11):9103–16. doi: 10.3390/cimb45110571 (PMC10670481; doi:10.3390/cimb45110571)
Supplement: Supplementary file 1 [file cimb-45-00571-s001.zip › Table S3.pdf]

Table S3. Upregulated DEGs between wild type and cloned piglets.

Gene symbol	GenBank accession number	Fold change	*P-value*	*FDR correction p-value*
NR4A3	NM_214247	12.83	1.11E-21	2.19E-19
PTX3	NM_001244783	12.68	3.60E-17	4.21E-15
CITED1	NM_001037964	8.04	3.11E-11	1.30E-09
KLF15	NM_001134349	6.71	2.04E-17	2.57E-15
FOS	NM_001123113	6.06	7.08E-15	5.59E-13
HSPA6	NM_001123127	5.80	1.44E-09	3.86E-08
CSF3	NM_213842	5.66	9.00E-11	3.35E-09
ACTC1	NM_001170517	5.44	1.15E-15	1.04E-13
FBXO32	NM_001044588	5.09	9.72E-11	3.53E-09
GADD45A	NM_001044599	5.02	1.49E-24	3.93E-22
PTGS2	NM_214321	4.91	5.89E-13	3.65E-11
STC1	NM_001103212	4.89	7.39E-35	5.84E-32
MYOZ2	NM_001044593	4.85	3.40E-17	4.14E-15
PLAGL1	NM_001044545	4.62	2.85E-09	7.19E-08
MSI1	NM_001315570	4.55	6.65E-10	1.95E-08
SFRP2	NM_001244395	4.51	4.48E-09	1.05E-07
PNMT	NM_001123164	4.30	4.86E-16	4.96E-14
AMZ1	NM_001195325	4.24	1.11E-07	1.87E-06
CXCL2	NM_001001861	4.15	2.13E-05	1.91E-04
AWN	NM_213829	4.14	8.63E-06	9.00E-05
LEPR	NM_001024587	4.11	1.71E-07	2.74E-06
SOCS3	NM_001123196	4.08	1.38E-11	6.33E-10
PPARGC1A	NM_213963	3.97	4.70E-06	5.36E-05
BHLHE40	NM_001245010	3.90	8.90E-34	5.63E-31
RBP5	NM_001145223	3.84	7.04E-08	1.26E-06
ADORA3	NM_001243435	3.80	3.70E-09	8.95E-08
CCL26	NM_001078665	3.79	1.00E-05	1.01E-04
FKBP5	NM_001315611	3.77	6.70E-31	3.53E-28
SERPINE1	NM_213910	3.76	6.00E-08	1.10E-06
CCR7	NM_001001532	3.74	1.15E-04	8.09E-04
RGS2	NM_001044600	3.69	9.37E-11	3.44E-09
SLC5A4	NM_214182	3.65	5.38E-08	1.00E-06
HMOX2	NM_001244412	3.58	1.12E-15	1.04E-13
GPR84	NM_001258301	3.51	2.03E-04	1.33E-03
CATSPER4	NM_001244280	3.50	1.09E-08	2.30E-07
HAPLN1	NM_001004028	3.47	2.90E-06	3.47E-05
ULBP1	NM_001004035	3.46	1.46E-06	1.84E-05
GALNT15	NM_001206447	3.41	1.82E-09	4.80E-08
DDIT4	NM_001243452	3.37	1.24E-30	5.59E-28
VCAM1	NM_213891	3.35	6.03E-22	1.27E-19
ZFP36	NM_001168419	3.32	7.07E-11	2.69E-09

NNAT	NM_001122990	3.32	1.89E-04	1.25E-03
CYP4F55	NM_001244636	3.31	8.51E-08	1.49E-06
GABARAPL1	NM_001190287	3.22	2.67E-25	8.43E-23
SLC30A2	NM_001139475	3.21	5.60E-10	1.70E-08
STC2	NM_001110173	3.15	2.21E-09	5.76E-08
NPPC	NM_001008482	3.15	5.15E-05	4.02E-04
GALP	NM_001348949, NM_213825	3.14	1.14E-04	8.03E-04
CPA1	NM_214244	3.10	9.31E-06	9.55E-05
BGLAP	NM_001164004	3.08	4.07E-06	4.69E-05
SPP1	NM_214023	3.08	9.50E-10	2.68E-08
HSD17B1	NM_001128472	3.06	1.60E-06	1.97E-05
DLK2	NM_001128466	3.05	2.59E-04	1.64E-03
EIF4EBP1	NM_001244225	2.99	9.16E-18	1.32E-15
CBLN4	NM_001123092	2.98	2.18E-05	1.94E-04
FAAH	NM_213914	2.88	1.20E-07	1.99E-06
PTN	NM_214336	2.87	4.15E-06	4.77E-05
WIF1	NM_001315718	2.81	6.24E-06	6.77E-05
CISH	NM_001315788	2.79	2.67E-12	1.41E-10
ADM	NM_214107	2.77	2.79E-07	4.22E-06
RTL1	NM_001134358	2.75	2.25E-05	2.00E-04
NFKBIZ	NM_001315599	2.74	7.49E-04	4.04E-03
RPL37	NM_001243603	2.71	1.68E-15	1.48E-13
KLF14	NM_001134348	2.71	3.90E-04	2.32E-03
RASGEF1B	NM_001195367	2.68	2.48E-13	1.67E-11
CAV3	NM_001037149	2.68	1.49E-05	1.41E-04
MYBPH	NM_001033014	2.68	3.56E-05	2.96E-04
LHX8	NM_001166315	2.67	8.78E-06	9.13E-05
IVNS1ABP	NM_001161649	2.66	2.48E-11	1.07E-09
DLK1	NM_001048187, NM_001126101	2.62	8.70E-08	1.51E-06
HSP70.2	NM_213766	2.62	1.55E-04	1.04E-03
MSR1	NM_001243874	2.58	1.92E-05	1.75E-04
SLC11A1	NM_213821	2.56	5.16E-04	2.94E-03
TNF	NM_214022	2.56	5.18E-03	1.98E-02
MYO7A	NM_001099928	2.53	6.71E-04	3.69E-03
DUSP1	NM_001256075	2.51	3.71E-09	8.95E-08
MIR24-2	NR_038538	2.49	1.98E-03	9.18E-03
MYF5	NM_001278775	2.45	1.41E-05	1.35E-04
ITIH3	NM_001206349	2.43	3.11E-05	2.64E-04
LSAMP	NM_001244697	2.42	7.12E-05	5.34E-04
AKR1CL1	NM_001038626	2.41	2.01E-02	6.08E-02
F3	NM_213785	2.40	2.08E-03	9.54E-03
SOCS1	NM_001204768	2.40	1.81E-08	3.64E-07
EDN1	NM_213882	2.40	5.93E-08	1.10E-06
RNF39	NM_001128446	2.39	3.40E-05	2.85E-04
TGFBR3	NM_214272	2.39	8.39E-04	4.41E-03

TNC	NM_214230	2.38	1.58E-03	7.50E-03
LOC100514340	NM_001244954	2.38	4.69E-04	2.72E-03
ADAMTS1	NM_001144843	2.37	4.59E-07	6.48E-06
SDC4	NM_214284	2.37	2.34E-05	2.06E-04
SMAD7	NM_001244175	2.36	2.64E-07	4.03E-06
PPARA	NM_001044526	2.35	5.36E-07	7.49E-06
LRRC2	NM_001128484	2.34	9.56E-07	1.24E-05
SAT1	NM_214358	2.33	2.54E-11	1.08E-09
KLF4	NM_001031782	2.30	6.89E-04	3.78E-03
PROX1	NM_001128490	2.28	4.83E-03	1.89E-02
TLR5	NM_001348771	2.27	5.19E-05	4.04E-04
MYC	NM_001005154	2.25	4.47E-05	3.58E-04
TNNI3	NM_001098599	2.24	4.62E-04	2.69E-03
RPS29	NM_001001633	2.24	2.39E-09	6.17E-08
LOC100511639	NM_001243872, NM_001243873	2.24	9.70E-05	6.95E-04
PTGFR	NM_214059	2.22	1.84E-04	1.22E-03
RGCC	NM_001244227	2.22	1.30E-10	4.45E-09
ABCA1	NM_001317080	2.21	1.50E-07	2.46E-06
SLC25A36	NM_001244793	2.21	4.30E-08	8.15E-07
YDJC	NM_001243562	2.19	3.96E-08	7.59E-07
LRPAP1	NM_001113436	2.19	1.53E-12	8.50E-11
EDN3	NM_001098582	2.19	1.27E-02	4.19E-02
RPS17	NM_001001634	2.19	3.11E-08	6.11E-07
ACKR1	NM_001244095	2.18	7.66E-04	4.10E-03
XIRP1	NM_001143928	2.18	2.98E-06	3.55E-05
CPEB2	NM_001185049	2.17	4.84E-04	2.78E-03
RPL22	NM_213987	2.16	8.63E-10	2.46E-08
FOSL2	NM_001315740	2.16	3.63E-05	3.00E-04
ALB	NM_001005208	2.15	1.27E-02	4.19E-02
TNMD	NM_001099934	2.14	2.67E-03	1.17E-02
GNMT	NM_001110419	2.14	6.89E-08	1.24E-06
C1QTNF7	NM_001143702	2.13	1.99E-05	1.81E-04
ANG	NM_001044573	2.13	6.48E-05	4.90E-04
RAB9A	NM_001244747	2.13	4.00E-11	1.60E-09
ZNF217	NM_001123217	2.12	6.84E-06	7.33E-05
FST	NM_001003662	2.12	8.06E-05	5.91E-04
SGCE	NM_001144124	2.11	1.81E-06	2.22E-05
NFKBIA	NM_001005150	2.10	4.10E-03	1.66E-02
WNT4	NM_001170828	2.10	2.92E-07	4.38E-06
RHOF	NM_001123190	2.09	2.05E-03	9.43E-03
ODC1	NM_001122983	2.09	2.10E-04	1.37E-03
TGIF1	NM_001244288, NM_001244289, NM_001244290	2.09	1.08E-24	3.11E-22
HBEGF	NM_214299	2.08	5.26E-03	2.01E-02
DES	NM_001001535	2.08	2.12E-07	3.32E-06

KLF10	NM_001134344	2.07	1.07E-07	1.84E-06
NDUFA4L2	NM_001205377	2.06	5.56E-03	2.10E-02
POLD2	NM_001244996	2.05	2.47E-07	3.80E-06
JAML	NM_001244730	2.05	4.03E-03	1.64E-02
IGF1R	NM_214172	2.04	1.57E-06	1.95E-05
EIF2C3	NM_001194974	2.01	3.08E-09	7.72E-08
CLK1	NM_001123099	2.01	5.94E-06	6.51E-05
RPS18	NM_213940	2.01	7.74E-05	5.74E-04
DMP1	NM_001129953	2.01	1.30E-05	1.26E-04
