# Supplementary material for: Characterization of Enlarged Tongues in Cloned Piglets
Source: Curr Issues Mol Biol. 2023 Nov 14;45(11):9103–16. doi: 10.3390/cimb45110571 (PMC10670481; doi:10.3390/cimb45110571)
Supplement: Supplementary file 1 [file cimb-45-00571-s001.zip › Table S4.pdf]

Table S4. Downregulated DEGs between wild type and cloned piglets.

Gene symbol	GenBank accession number	Fold change	*P-value*	*FDR correction p-value*
OAS1	NM_214303	-32.42	1.02E-53	3.21E-50
GPD1	NM_001190240	-15.09	3.97E-52	6.27E-49
C4BPA	NM_213942	-10.33	7.60E-13	4.53E-11
LTF	NM_214362	-8.82	4.00E-15	3.24E-13
FBP2	NM_001167632	-8.66	9.44E-14	6.48E-12
INHA	NM_214189	-7.68	7.71E-12	3.87E-10
PYY	NM_001256528	-7.08	4.94E-12	2.56E-10
NPG4	NM_213863	-6.87	1.62E-11	7.30E-10
DMBT1	NM_001048188	-6.48	6.95E-07	9.47E-06
MLPH	NM_001098594	-6.43	1.15E-10	3.98E-09
PG-2	NM_001129966	-6.07	3.69E-08	7.16E-07
PGLYRP1	NM_001001260	-5.99	1.32E-11	6.21E-10
PR39	NM_214450	-5.53	3.17E-08	6.18E-07
KCNS3	NM_001044596	-5.47	6.91E-23	1.68E-20
LDHA	NM_001172363	-5.02	9.67E-27	3.82E-24
CLCA1	NM_214148	-4.81	2.82E-05	2.44E-04
SNCG	NM_001103211	-4.71	1.90E-17	2.51E-15
FUT2	NM_214069	-4.68	7.61E-08	1.35E-06
NPG1	NM_001123149	-4.63	1.12E-05	1.11E-04
HP	NM_214000	-4.55	2.54E-09	6.47E-08
CRHR2	NM_001144118	-4.44	6.88E-19	1.09E-16
HBB	NM_001144841	-4.43	8.45E-13	4.86E-11
SLC9A2	NM_001100189	-4.43	1.12E-19	2.07E-17
MYH4	NM_001123141	-4.43	7.28E-16	7.19E-14
NPY1R	NM_214288	-4.41	1.35E-08	2.82E-07
TSHR	NM_214297	-4.40	1.49E-08	3.07E-07
AVPR2	NM_214232	-4.39	1.61E-09	4.27E-08
CRACR2B	NM_001243838	-4.34	1.10E-10	3.92E-09
CD4	NM_001001908	-4.25	4.02E-06	4.65E-05
PMAP-23	NM_001129976	-4.25	2.13E-05	1.91E-04
GNLY	NM_001278755	-4.13	5.67E-09	1.31E-07
ADRA1D	NM_001123073	-4.02	1.05E-09	2.94E-08
MPEG1	NM_001267892	-3.85	8.48E-09	1.89E-07
SLC28A3	NM_001244637	-3.81	5.00E-05	3.94E-04
CCDC42	NM_001244077	-3.77	1.89E-07	2.97E-06
RSPO3	NM_001315656	-3.77	1.33E-10	4.52E-09
HTRA3	NM_001195343	-3.74	1.26E-11	6.01E-10
AQP5	NM_001110424	-3.69	9.35E-05	6.76E-04
NEFL	NM_001244331	-3.68	4.45E-05	3.58E-04
PEBP4	NM_001162888	-3.65	8.61E-11	3.24E-09
RDH16	NM_001243866	-3.65	1.56E-06	1.94E-05

CCRL2	NM_001001617	-3.65	5.05E-13	3.19E-11
BCHE	NM_001357509	-3.61	1.17E-35	1.24E-32
GYS1	NM_001195508	-3.54	7.95E-13	4.65E-11
KCNAB1	NM_001105294	-3.51	1.21E-26	4.23E-24
CHGB	NM_214081	-3.51	1.04E-05	1.04E-04
IGSF6	NM_001243387	-3.50	1.95E-10	6.49E-09
MSTN	NM_214435	-3.50	8.35E-05	6.10E-04
MTTP	NM_214185	-3.49	4.08E-05	3.31E-04
OAS2	NM_001031796	-3.46	6.64E-10	1.95E-08
PI16	NM_001243527	-3.46	8.54E-10	2.45E-08
LOC100513601	NM_001246242	-3.44	8.73E-04	4.56E-03
NCEH1	NM_001243484	-3.40	3.04E-10	9.81E-09
PFKM	NM_001044550	-3.38	6.36E-11	2.51E-09
FADS1	NM_001113041	-3.34	4.55E-16	4.79E-14
MT3	NM_214056	-3.33	2.20E-05	1.96E-04
SLA-DRB1	NM_001113695	-3.33	2.87E-10	9.35E-09
CASQ1	NM_001243269	-3.31	6.14E-13	3.73E-11
HOMER1	NM_001243811	-3.30	3.11E-10	9.93E-09
SYN1	NM_001141988	-3.29	1.99E-14	1.43E-12
G0S2	NM_001286804	-3.24	2.26E-19	3.77E-17
ACY1	NM_213896	-3.22	1.13E-18	1.70E-16
AOX1	NM_001308473	-3.17	2.71E-04	1.70E-03
KRT8	NM_001159615	-3.15	8.61E-07	1.14E-05
MYOC	NM_213986	-3.13	1.05E-08	2.23E-07
TMOD4	NM_001243294	-3.12	9.95E-09	2.14E-07
PLBD1	NM_001244629	-3.09	3.32E-09	8.18E-08
METTL11B	NM_001243649	-3.08	6.75E-05	5.09E-04
AQP7	NM_001113438	-3.06	1.22E-09	3.32E-08
SCIN	NM_001244731	-3.03	2.45E-04	1.56E-03
GALNT16	NM_001244918	-3.02	1.70E-10	5.70E-09
MYL3	NM_001278773	-3.01	4.66E-04	2.71E-03
AQP11	NM_001112682	-3.00	2.87E-07	4.32E-06
TFRC	NM_214001	-2.99	1.21E-14	9.08E-13
LHB	NM_214080	-2.98	1.01E-05	1.02E-04
C13H21orf62	NM_001163696	-2.98	4.00E-10	1.23E-08
RGS5	NM_214356	-2.98	4.07E-13	2.68E-11
EGF	NM_214020	-2.97	9.92E-09	2.14E-07
OBP2B	NM_213856	-2.97	3.85E-04	2.30E-03
CCL19	NM_001170516	-2.96	3.69E-05	3.04E-04
SLC37A4	NM_001199719	-2.94	2.84E-15	2.36E-13
GGT1	NM_214030	-2.92	7.72E-05	5.74E-04
GPI	NM_214330	-2.92	2.30E-11	1.02E-09
AMPD1	NM_001123076	-2.92	2.56E-15	2.19E-13
CNN1	NM_213878	-2.92	3.78E-06	4.40E-05
ADAP2	NM_001195323	-2.91	6.46E-10	1.93E-08
RHCE	NM_214378	-2.89	3.44E-04	2.08E-03

TDRD10	NM_001246198	-2.89	2.42E-08	4.77E-07
TCF19	NM_001167591	-2.86	3.67E-10	1.15E-08
FITM2	NM_001128460	-2.85	1.70E-07	2.74E-06
CKMT2	NM_001044551	-2.84	5.68E-06	6.37E-05
GP9	NM_001141989	-2.84	5.10E-05	4.00E-04
FCN2	NM_213868	-2.81	4.57E-07	6.48E-06
PGM1	NM_001246318	-2.80	2.55E-12	1.37E-10
CAMK2G	NM_214193	-2.78	3.89E-22	8.77E-20
PGK1	NM_001099932	-2.78	1.68E-17	2.31E-15
STARD4	NM_001143726	-2.74	3.08E-03	1.33E-02
CREB3L4	NM_001123102	-2.74	3.04E-06	3.61E-05
ATP2A2	NM_213865	-2.72	5.37E-12	2.74E-10
MARCH2	NM_001244915	-2.71	3.44E-09	8.44E-08
AQP1	NM_214454	-2.66	1.76E-07	2.78E-06
TPI1	NM_001037151	-2.65	1.66E-14	1.22E-12
TMEM106A	NM_001244554	-2.63	6.66E-11	2.57E-09
PHKG1	NM_001293144	-2.61	4.76E-09	1.11E-07
ATP2A1	NM_001204393	-2.58	4.01E-09	9.47E-08
CALML4	NM_001244619	-2.56	3.96E-06	4.60E-05
NTF4	NM_001243713	-2.56	5.72E-09	1.31E-07
BCAS1	NM_001110175	-2.55	6.89E-06	7.36E-05
CEND1	NM_213866	-2.54	1.63E-04	1.09E-03
CLGN	NM_001243207	-2.52	1.54E-03	7.36E-03
CACNG1	NM_001044605	-2.51	1.44E-05	1.37E-04
PRKAB2	NM_001243683	-2.50	2.32E-11	1.02E-09
FLVCR1	NM_001142846	-2.49	5.04E-07	7.08E-06
CLIC5	NM_001198923	-2.48	3.55E-11	1.44E-09
SEC14L2	NM_001198918	-2.48	1.09E-03	5.48E-03
FBP1	NM_213979	-2.46	1.40E-03	6.83E-03
HYAL1	NM_214441	-2.43	9.74E-05	6.97E-04
TBC1D10B	NM_001244423	-2.41	1.19E-11	5.88E-10
AKR1B1	NM_001001539	-2.39	5.66E-10	1.70E-08
PDYN	NM_001004040	-2.38	6.22E-04	3.47E-03
PHYHIPL	NM_001244581	-2.37	3.38E-03	1.43E-02
SLA-1	NM_001097431	-2.34	3.82E-04	2.28E-03
ATP6V1A	NM_001004042	-2.34	7.98E-08	1.40E-06
ENO3	NM_001044527	-2.34	1.19E-07	1.98E-06
IFITM3	NM_001201382	-2.34	1.26E-02	4.18E-02
MUC4	NM_001206344	-2.32	2.81E-02	7.91E-02
ITGB1BP2	NM_001033009	-2.32	7.62E-09	1.71E-07
CYP2B22	NM_214413	-2.31	7.98E-04	4.24E-03
RNASEL	NM_001097512	-2.31	2.27E-03	1.03E-02
MYH1	NM_001104951	-2.30	2.06E-05	1.86E-04
P2RY11	NM_001204436	-2.29	6.97E-04	3.81E-03
ARHGDIB	NM_001244240	-2.29	1.31E-09	3.53E-08
OLR1	NM_213805	-2.27	7.63E-03	2.76E-02

RBP1	NM_001031789	-2.23	3.35E-03	1.42E-02
CYSLTR2	NM_214130	-2.20	4.74E-06	5.39E-05
CLDN10	NM_001243444	-2.20	5.80E-04	3.28E-03
KCNJ2	NM_214151	-2.19	4.41E-05	3.56E-04
GFAP	NM_001244397	-2.19	4.37E-03	1.75E-02
CACNA2D1	NM_214183	-2.19	5.13E-04	2.93E-03
LOC733579	NM_001044532	-2.18	1.07E-06	1.38E-05
TLR6	NM_213760	-2.18	2.40E-09	6.17E-08
IL34	NM_001285975	-2.18	4.48E-05	3.59E-04
TOB1	NM_001123205	-2.17	9.84E-09	2.14E-07
IFITM2	NM_001246214	-2.17	9.31E-09	2.06E-07
CCND2	NM_214088	-2.15	3.88E-09	9.21E-08
IGFBP7	NM_001163801	-2.15	1.68E-08	3.40E-07
ANKRD23	NM_001315720	-2.15	1.18E-04	8.27E-04
SAL1	NM_213814	-2.15	2.74E-02	7.80E-02
MYL9	NM_001244472	-2.14	9.90E-06	1.01E-04
AKR1C4	NM_001123075	-2.14	4.54E-02	1.14E-01
SLC31A1	NM_214100	-2.14	4.14E-07	5.95E-06
ALDH5A1	NM_001244467	-2.13	5.56E-07	7.74E-06
HMGCS1	NM_001252215	-2.13	1.37E-02	4.44E-02
RAD54L	NM_001123183	-2.12	6.53E-07	8.93E-06
ARF2	NM_001145220	-2.12	5.58E-14	3.92E-12
TRIM26	NM_001123209	-2.12	1.46E-06	1.84E-05
ATP5F1A	NM_001185142	-2.11	1.39E-08	2.89E-07
SLC16A1	NM_001128445	-2.11	8.81E-07	1.17E-05
ORAI1	NM_001173519	-2.11	8.54E-15	6.58E-13
HSPA8	NM_001243907	-2.11	7.33E-10	2.12E-08
CYP7A1	NM_001005352	-2.10	3.52E-03	1.47E-02
CA4	NM_001243920	-2.09	5.98E-04	3.36E-03
GOT2	NM_213928	-2.09	1.14E-10	3.98E-09
SLPI	NM_213870	-2.09	1.94E-03	9.03E-03
NLRC5	NM_001278781	-2.09	3.62E-04	2.17E-03
HTR2B	NM_001164019	-2.09	1.09E-03	5.48E-03
MAOB	NM_001001864	-2.09	1.15E-02	3.90E-02
DNAJC6	NM_001145378	-2.08	8.39E-04	4.41E-03
LCK	NM_001143713	-2.08	7.41E-04	4.01E-03
SERTM2	NM_001354500	-2.07	7.88E-05	5.80E-04
ZC3HAV1	NM_001177489	-2.07	1.75E-07	2.78E-06
PTHLH	NM_213916	-2.07	1.60E-02	5.00E-02
B4GALNT2	NM_001244330	-2.07	4.01E-05	3.26E-04
IMPA1	NM_214216	-2.06	1.09E-09	3.02E-08
GOT1	NM_213927	-2.06	3.30E-05	2.78E-04
TAP1	NM_001044581	-2.06	5.02E-13	3.19E-11
SLC6A6	NM_001319102	-2.06	1.92E-03	8.98E-03
MLLT11	NM_001244607	-2.06	9.70E-06	9.90E-05
GAL3ST1	NM_001244429	-2.05	3.98E-03	1.63E-02

KAZALD1	NM_001244551	-2.04	1.25E-04	8.63E-04
RETN	NM_213783	-2.04	3.28E-02	8.97E-02
TF	NM_001244653	-2.03	1.83E-02	5.61E-02
PRKG1	NM_001044574	-2.03	1.69E-03	8.01E-03
SLC26A6	NM_001012298	-2.03	3.46E-05	2.87E-04
MGST1	NM_214300	-2.03	3.16E-09	7.86E-08
ITGB6	NM_001097423	-2.02	6.67E-04	3.68E-03
PSMB9	NM_001037961	-2.02	1.45E-08	3.00E-07
HMGCR	NM_001122988	-2.02	8.55E-03	3.04E-02
B3GNT5	NM_214327	-2.02	6.57E-04	3.63E-03
CD247	NM_214155	-2.02	3.96E-08	7.59E-07
HAAO	NM_001244941	-2.02	4.64E-05	3.68E-04
LOC100153094	NM_001315568	-2.02	1.16E-05	1.14E-04
UGT8	NM_001315662	-2.01	4.38E-03	1.75E-02
SUV39H2	NM_001039747	-2.01	3.11E-07	4.64E-06
NFE2	NM_001185152	-2.00	2.06E-04	1.34E-03
SELENOT	NM_001163408	-2.00	1.56E-08	3.18E-07
MMD	NM_001044595	-2.00	1.75E-05	1.61E-04
