# Supplementary material for: Characterization of Enlarged Tongues in Cloned Piglets
Source: Curr Issues Mol Biol. 2023 Nov 14;45(11):9103–16. doi: 10.3390/cimb45110571 (PMC10670481; doi:10.3390/cimb45110571)
Supplement: Supplementary file 1 [file cimb-45-00571-s001.zip › Table S5.pdf]

Table S5. Analysis of enriched biological process GO Terms for DEGs.

GO term	Description	Fold change	enrichment FDR
GO:0003012	Muscle system process	6.20	4.25E-08
GO:0048878	Chemical homeostasis	3.90	3.01E-10
GO:0051241	Negative regulation of multicellular organismal proc.	3.44	2.72E-07
GO:0051707	Response to other organism	3.39	9.14E-08
GO:0043207	Response to external biotic stimulus	3.39	9.14E-08
GO:1901700	Response to oxygen-containing compound	3.16	9.98E-08
GO:0044419	Biological process involved in interspecies interaction between organisms	3.14	1.79E-07
GO:0042592	Homeostatic process	3.12	4.49E-10
GO:0051239	Reg. of multicellular organismal process	3.07	4.66E-16
GO:0009605	Response to external stimulus	2.97	2.55E-12
GO:0009888	Tissue development	2.79	1.27E-08
GO:0032879	Reg. of localization	2.76	7.29E-12
GO:0070887	Cellular response to chemical stimulus	2.63	3.52E-11
GO:0016310	Phosphorylation	2.56	2.72E-07
GO:0002376	Immune system process	2.53	6.87E-09
GO:0071310	Cellular response to organic substance	2.51	1.24E-07
GO:0065009	Regulation of molecular function	2.43	7.05E-08
GO:0010033	Response to organic substance	2.39	5.24E-08
GO:0035556	Intracellular signal transduction	2.33	3.11E-07
