# Supplementary material for: Characterization of Enlarged Tongues in Cloned Piglets
Source: Curr Issues Mol Biol. 2023 Nov 14;45(11):9103–16. doi: 10.3390/cimb45110571 (PMC10670481; doi:10.3390/cimb45110571)
Supplement: Supplementary file 1 [file cimb-45-00571-s001.zip › Table S6.pdf]

Table S6. Analysis of enriched KEGG pathway for DEGs.

GO term	Description	Fold change	enrichment FDR
ssc00030	Pentose phosphate pathway	12.75	9.1.E-04
ssc00010	Glycolysis / Gluconeogenesis	9.52	8.5.E-05
ssc04922	Glucagon signaling pathway	8.83	1.6.E-06
ssc04612	Antigen processing and presentation	8.74	1.1.E-03
ssc00330	Arginine and proline metabolism	8.57	1.2.E-03
ssc05134	Legionellosis	8.08	1.4.E-03
ssc04066	HIF-1 signaling pathway	7.93	2.7.E-06
ssc04924	Renin secretion	7.57	9.1.E-04
ssc05410	Hypertrophic cardiomyopathy	7.47	1.3.E-04
ssc01230	Biosynthesis of amino acids	7.04	1.1.E-03
ssc04152	AMPK signaling pathway	6.71	4.4.E-05
ssc04260	Cardiac muscle contraction	6.33	1.6.E-03
ssc01200	Carbon metabolism	5.84	6.9.E-04
ssc04024	CAMP signaling pathway	5.78	1.6.E-06
ssc04910	Insulin signaling pathway	5.37	5.7.E-04
ssc05418	Fluid shear stress and atherosclerosis	4.98	1.3.E-03
ssc04630	JAK-STAT signaling pathway	4.73	1.1.E-03
ssc05417	Lipid and atherosclerosis	4.11	1.3.E-03
ssc04080	Neuroactive ligand-receptor interaction	3.58	4.2.E-04
ssc01100	Metabolic pathways	2.35	2.7.E-06
